# A Novel Homozygous Nonsense Mutation p.Cys366* in the WNT10B Gene Underlying Split-Hand/Split Foot Malformation in a Consanguineous Pakistani Family

**DOI:** 10.3389/fped.2019.00526

**Published:** 2020-01-09

**Authors:** Amjad Khan, Rongrong Wang, Shirui Han, Muhammad Umair, Mohammad A. Alshabeeb, Muhammad Ansar, Wasim Ahmad, Manal Alaamery, Xue Zhang

**Affiliations:** ^1^McKusick-Zhang Center for Genetic Medicine, Institute of Basic Medical Sciences Chinese Academy of Medical Sciences, School of Basic Medicine Peking Union Medical College, Beijing, China; ^2^The Research Center for Medical Genomics, China Medical University, Shenyang, China; ^3^Developmental Medicine Department, King Abdullah International Medical Research Center (KAIMRC), King Saud Bin Abdulaziz University for Health Sciences, Ministry of National Guard-Health Affairs (MNGHA), Riyadh, Saudi Arabia; ^4^Medical Genomics Research Department, King Abdullah International Medical Research Center (KAIMRC), King Saud Bin Abdulaziz University for Health Science, Ministry of National Guard-Health Affairs (MNGHA), Riyadh, Saudi Arabia; ^5^Department of Biochemistry, Faculty of Biological Sciences, Quaid-i-Azam University, Islamabad, Pakistan

**Keywords:** Pakistani family, SHFM, autosomal recessive mode, gene variant, non-sense mutation

## Abstract

Split hand/split foot malformation (SHFM) or ectrodactyly is characterized by a deep median cleft of the hand or foot, hypoplasia or aplasia of the metacarpals, metatarsals, and phalanges. It is a clinically and genetically heterogeneous group of limb malformations. This study aimed to identify the pathogenic variant in a consanguineous Pakistani family with autosomal recessive SHFM. Peripheral blood samples were obtained, DNA was extracted, *WNT10B* coding and noncoding regions were PCR amplified and Sanger sequencing was performed using workflow suggested by Thermo Fisher Scientific. A novel homozygous nonsense variant (c.1098C>A; p.Cys366*) was identified in the *WNT10B* gene in the index patients, which probably explains SHFM type 6 in this family in comparison with similar data from the literature.

## Introduction

Split-hand/split-foot malformation (SHFM; OMIM:225300) or ectrodactyly is a rare limb developmental disorder. It is characterized by a deep median cleft of the hand or foot corresponding to the central rays of the autopod (1, 2). Phalangeal aplasia and hypoplasia of metacarpals and metatarsals are also signature features of SHFM. It has an incidence rate of ~1 in 90,000 live births (3, 4). Both individuals and families with SHFM can have a highly variable presentation ranging from minor clefting at the central ray to severe, lobster claw-like deformity (5, 6). It can exist as an isolated entity or as a complex syndrome. Few patients have been reported exhibiting craniofacial defects, ectodermal dysplasia or intellectual disability (5–7). Typically, SHFM is inherited as an autosomal dominant trait with incomplete penetrance; however, several cases have been reported with an autosomal recessive or X-linked inheritance pattern (7–9). To date, eight types of non-syndromic SHFM were described and mapped to different chromosomes. Four autosomal dominant types have been mapped to chromosomes 2q31 (SHFM-5; MIM 606708), 3q27 (SHFM-4; MIM 605289), 7q21 (SHFM-1; MIM 183600), 10q24 (SHFM-3; MIM 246560), and the X-linked form to Xq26.3 (SHFM-2; MIM 313350) (10–15). The autosomal recessive SHFM-6 (MIM 225300) was mapped to chromosome 12q13.12 harboring the wingless-type MMTV integration site family member 10 (*WNT10B*, MIM 601906) (8, 9). Mutations in several genes have been associated with SHFM including *TP63, DLX5, DLX6, ZAK, FGFR1, EPS15L1*, and *WNT10B;* however, the clinical features are often indistinguishable (16–18). In the zebrafish, the expression of *TP63, DLX5, DLX6, FGFR1*, and *WNT10B* was shown in the fin's apical ectodermal ridge (AER) cells (16). A similar pattern was also observed in the mouse limbs (19).

In this study, we described a consanguineous family of Pakistani origin showing SHFM with recessive inheritance. Sanger sequencing of *WNT10B* was used to possibly detect a novel homozygous nonsense mutation that co-segregating with SHFM phenotype within the studied family. We provided a brief phenotype comparison of this family to other cases described in the literature.

## Case Presentation

### Methods

#### Ethics and Consent Approval

The study design and protocol were approved by the Institutional Review Board (IRB) at Quaid-i-Azam University Islamabad Pakistan, the Ethical Review Committee (ERC) of Peking Union Medical College (Beijing, China), and China Medical University (Shenyang, China). For minors, written informed consent was signed by their parents.

### Patients and DNA Sample Collection

**Figure 1:**
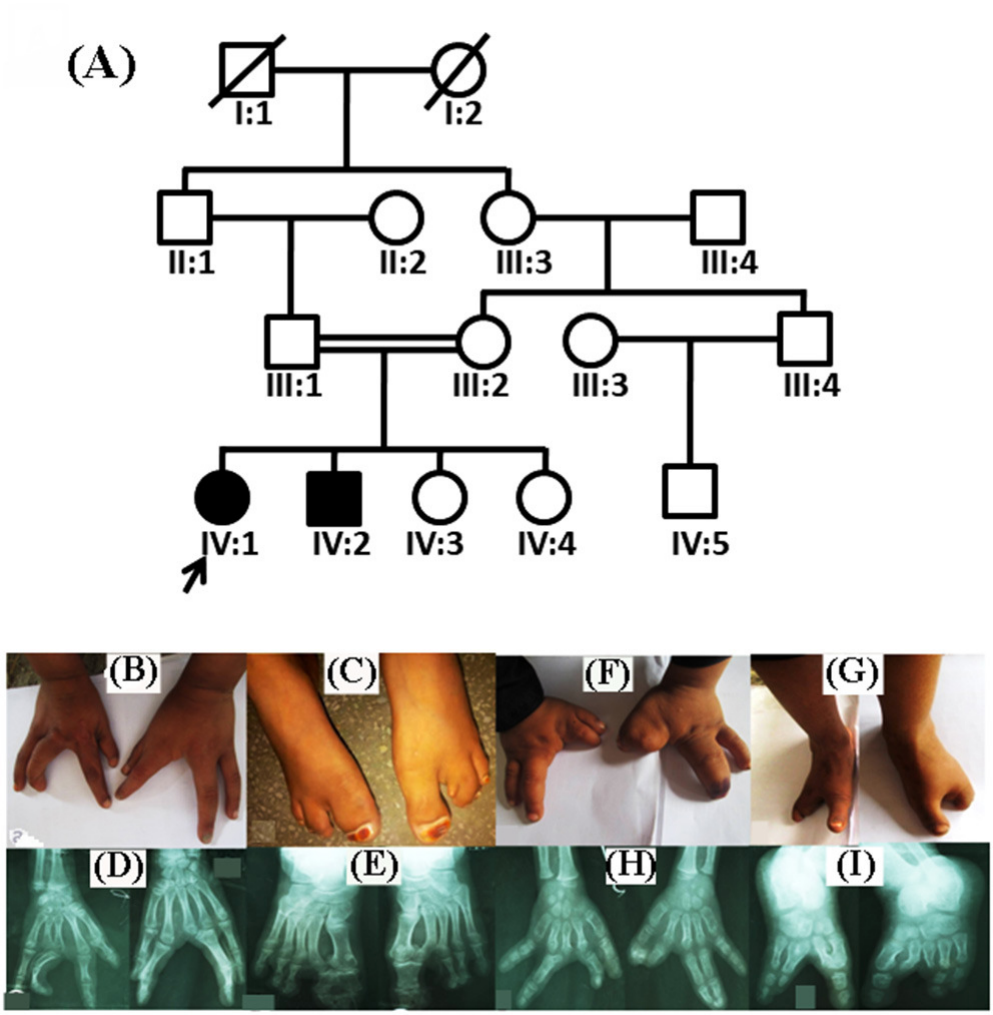
Pedigree analysis and clinical features of split hand/foot malformation (SHFM) observed in the family. **(A)** A consanguineous pedigree showing two affected members (IV:1 and IV:2) in the fourth generation. Affected individuals in the pedigree are shown with shaded symbols and unaffected with open symbols. **(B,C)** 14 years old affected individual (IV:1), showing fusion of proximal phalanx with a median cleft in both hands, and clinodactyly in the left hand. Feet showing complex pre axial syndactyly. **(D,E)** Radiographic examination of the affected individual (IV:1) revealed median cleft in both hands, aplasia, with complex syndactyly, while feet showed complex preaxail syndactyly of the toes and 1st and 2nd metatarsals. **(F,G)** Affected individual (IV:2) having aplasia in both hands and feet, missing digits and complex syndactyly, feet exhibiting typical median cleft phenotypes. **(H,I)** Radiographs of the same affected individual revealed missing digits in both hands and feet, complex preaxial and postaxial syndactyly.

The two index patients (IV:1, IV:2) of Pakistani origin were children from a consanguineous marriage (Figure 1A). Blood samples were collected from individuals III:1, III:2, IV:1, IV:2, and IV:3. The QIAquick DNA extraction kit (QIAamp, Qiagen, Valencia, CA, USA) was used for genomic DNA extraction. The DNA quantity and quality were assessed using nanodrop-2000 spectrophotometer (Thermo Scientific, Schaumburg, IL, USA).

### Sanger Sequencing of *WNT10B*

Genomic sequence of *WNT10B* gene, including exons, introns, 5′ untranslated region (UTR) and 3′ UTR, was retrieved from the University of California Santa Cruz genome database browser (UCSC; http://genome.ucsc.edu/). Oligonucleotide primer pairs of the five exons in WNT10B were designed with Gene Runner software (version 5.0.69 Beta; Hastings Software, Inc., Hastings, NY) (Table 1). For all family members, all coding and noncoding regions of the *WNT10B* was PCR-amplified in 20 μL reaction volume with 10 pMol of each primer pair and sequenced by Sanger's method after purification. Standard sequencing protocol was followed using BigDye^®^ Terminator v3.1 and Cycle Sequencing Kit (Applied Biosystems, USA). BioEdit tool was applied to analyze and detect mutation in sequenced data using NCBI GeneBank accession number [NG_023347.1] as a reference for alignment. In addition, 200 ethnically-matched control samples were sequenced to assess the allele frequency of the novel variant.

**Table 1:** Primers for *WNT10B* PCR amplification.

**Primers**	**Forward primer (5^**′**^-3^**′**^)**	**Reverse primer (5^**′**^-3^**′**^)**	**Product length (bp)**
*WNT10B-1*	ggagagggtgtgtgagagag	tggctctctatgcgtctctg	598
*WNT10B-2*	ctgaacccgcatcaagtctc	gtcggtgtttctatggcctg	187
*WNT10B-3*	caggccatagaaacaccgac	ctagggtaggagagcaggga	583
*WNT10B-4*	ttacctccaccatcacaccc	gcctctcaaactctaaccagg	471
*WNT10B-5*	ctccatttgtccctccctgt	ttccagggaccaagagtgac	669

## Results

### Clinical and Radiological Examinations

Both patients (IV:1 and IV:2) had a physical examination and X-rays performed by an orthopedic surgeon. Patients during the time of recruitment for genetic analysis were 14 (IV:1) and 12 (IV:2) years of age. Both patients of the family exhibited SHFM phenotype with involvement of hands and feet segregated in an autosomal recessive manner. Parents of the affected patients were normal and healthy. The patient IV:1 had complex syndactyly, clinodactyly, dysplastic hands and feet (Figures 1B–E). In contrast, the patient IV:2 had syndactyly in the left hand and both feet, aplasia of the radial ray of hand and hallux valgus deformity in the big toe. The distal phalanx of the middle finger was also missing. Photos of the hands and feet are shown in Figures 1F–I. No other dysmorphic features were observed. Both patients had an average intellect and were attending regular school classes with satisfactory performance.

#### Radiological Examinations

During the radiological evaluation, the patient IV:1 showed an absence of the first metacarpal and multiple phalanges in both hands. Both feet had deep midline cleft and syndactyly (Figures 1B–E). Radiographic examination of Patient IV:2 showed the absence of multiple metatarsals and phalanges in both feet (Figures 1F–I). Detailed clinical information of both patients were compared to previously reported SHFM cases as seen in Table 2.

**Table 2:** Clinical features and WNT10B variations detected in the present family and those families reported previously.

**DNA variation**	**Protein variation**	**Exon location**	**Major clinical phenotype**	**Ethnicity**
c.994C>T	p.Arg332Trp	5	Syndactyly, Postaxial syndactyly 3rd and 4th fingers with almost fused nail beds, clinodactyly in finger 5, polydactyly type 1.	Turkish (8)
c.986C>G	p.Thr329Arg	5	Pre-axial syndactyly of 1st and 2nd toe, post-axial syndactyly of 3rd and 4th toe, absence of 2nd toe, hypoplasia in 2nd digit, Agenesis of the distal ray at meta-carpophalangeal joint level, fixed flexion contracture of both 4th and 5th digit at the proximal interphalangeal joint level, pre-axial polydactyly type 1.	Pakistani (9)
c.1165_1168delAAGT c.300_306dupAGGGCGG	p.Lys388Glufs^*^36, p.Leu103Argfs^*^53	5 3	polydactyly, pre- axial syndactyly, campodactyly, dysplastic hands and cleft feet, hallux valgus deformity of big toe and rudimentary bud of lesser toes.	Pakistani (24)
c.460C>G	p.Gln154^*^	4	Pre-axial syndactyly of index finger, Mesoaxial type of syndactyly, cleft hand with the absence of a middle finger, Aplasia of the middle finger, missing central toes, missing of great thumb, hallux valgus deformities of big toe.	Pakistani (25)
c.300_306dupAGGGCGG	p.Leu103Argfs^*^53	3	Syndactyly of the 1st and 2nd toe, hypoplasia of 1st metacarpal, complex fusion of 3rd and 4th finger, bilateral hypoplasia and fusion of the 3rd and 4th finger, claw toe deformity.	Pakistani (25)
c.676C>T c.338-1G>C	p.Arg226^*^ –	4 3	Syndactyly, polydactyly, flexion contracture of the left index finger, dysplasia of the distal/middle phalanges of the right index finger, and dysplasia of the postaxial toes of both feet.	Saudi (12)
c.695_697delACA	p.Asn232del	4	Asymmetric longitudinal deficiency of the central rays of hands, absence of the middle and distal phalanges of the 3rd right and left fingers, delayed ossification of all carpal bones, asymmetric longitudinal deficiency of central rays of feet, fusion of the right 1st and 2nd metatarsals, absence of the left 2nd, 3rd, and 4th metatarsals and their corresponding phalanges, absence of the right 3rd metatarsal and 2nd, 3rd, and 4th proximal, middle, and distal phalanges.	Indian (26)
c.1098C>A	p.Cys366^*^	5	Proximal phalanx with median cleft in both hands, clinodactyly in left hand. Feet showing complex preaxial syndactyly, median cleft in both hands, aplasia, with complex syndactyly, complex pre-axial syndactyly of the toes and 1st and 2nd metatarsal, complex preaxial and post axial syndactyly in hand and feet.	This study

### Mutation Confirmation

**Figure 2:**
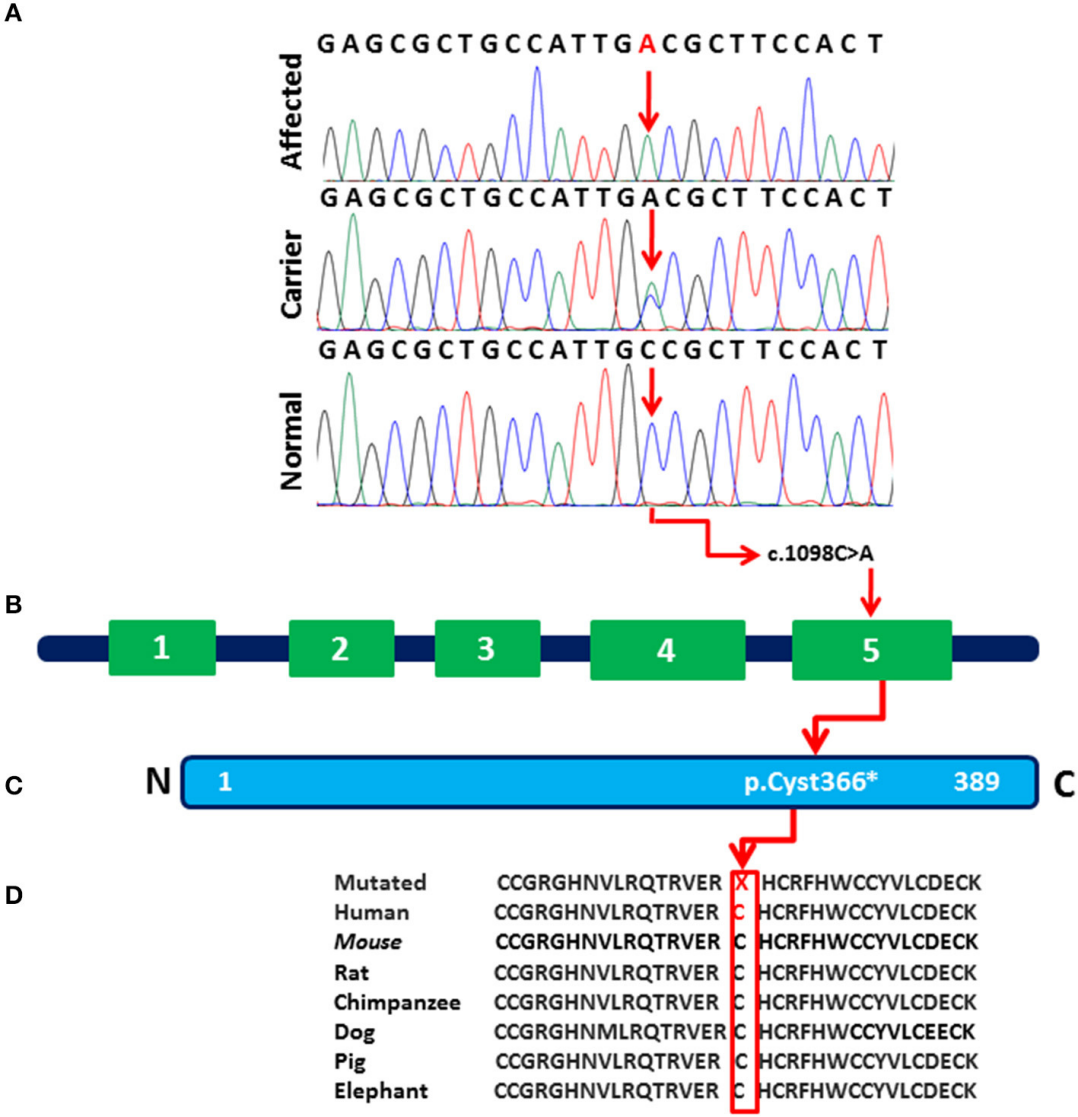
**(A)** Sequence chromatogram of the *WNT10B* gene showing a homozygous nonsense mutation (c.1098C>A; p.Cys366^*^) in affected individuals (IV:1 and IV:2), a heterozygous variant in (III:1, III:2, IV:3) and control reference in 200 ethnically-matched normal individuals. **(B)** The exon/intron structure of the *WNT10B* gene along with reported mutation causing SHFM phenotypes. Variant (c.1098C>A) marked with black color represents our index patients. **(C)** Variant c.1098C>A (p.Cys366^*^) detected in the present study affect the last region (amino acids 367–389) of the *WNT10B* domain. **(D)** The amino acids mutated in the affected individuals are conserved in different species.

Analysis of Sanger sequencing shown in Figure 2A identified a homozygous nonsense variant (c.1098C>A; p.Cys366*) in exon 5 of the *WNT10B* gene in both affected individuals (IV:1 and IV:2) of the family. Their parents were heterozygous for the same variant (Figure 2B). This variant leads to premature termination of the 389 amino acid protein in the main WNT domain (Figure 2C). Other vertebrate species indicated in Figure 1D share similar variant and an overall gene sequence.

### *In-silico* Analysis

The novelty and pathogenicity of the identified variant were predicted using different online *in silico* analysis tools including Polyphen-2 (http://genetics.bwh.harvard.edu/pph2/), Sorting Intolerant From Tolerant (SIFT, http://sift.jcvi.org/), Protein Variation Effect Analyzer (PROVEAN, http://provean.jcvi.org), Mutation Taster (http://www.mutationtaster.org/), Varsome (https://varsome.com/), and Combined Annotation Dependent Depletion (CADD, https://cadd.gs.washington.edu/). Finally, for the interpretation of variants, American College of Medical Genetics and Genomics (ACMG) 2015 guidelines were used (20) (Table 3). The variant (c.1098C>A; p.Cys366*) was not observed in gnomAD (https://gnomad.broadinstitute.org/), 1000 Genomes (http://www.1000genomes.org/), dbSNP (http://www.ncbi.nlm.nih.gov/SNP/), and in the Exome Aggregation Consortium (ExAC, http://exac.broadinstitute.org) and was not present in 200 ethnically-matched control individuals.

**Table 3:** Molecular information of homozygous variant on chromosome 12 of *WNT10B* family.

**Family ID**	**Family**
Affected individuals	Male,1Female
Transcript ID	NM_003394.4
Gene name	*WNT10B*
Chromosomal location	12q13.12
MIM number	601906
Chromosome position	Chr12:49359950
Nucleotide change	c. 1098C>A
Protein change	p.Cys366^*^
1000G_ALL	–
ExAC_Freq	–
LRT	0.000/Damaging
gnomAD	–
SIFT Score_pred	–
Polyphen2 Score_pred	–
Mutation Taster Score_pred	–
FATHMM_MKL	0.986/damaging
CADD Score	38/damaging
PROVEAN Score_pred	–
DANN	0.991/damaging
ACMG classification	Pathogenic
Variant status	Novel
Segregation with phenotype	Yes

## Discussion

In this study, we identified a novel homozygous nonsense variant c.1098C>A in *WNT10B* in a consanguineous family having SHFM. The nonsense variant (c.1098C>A; p.Cys366*) results in a Cysteine amino acid substitution to stop codon at a position 366 which results in a shorter protein formation (21). A number of families has been reported previously from Pakistan having different mutations in the *WNT10B* leading to SHFM phenotypes (22–24). Khan et al. (9), Aziz et al. (24), and Ullah et al. (25), studied several Pakistani consanguineous SHFM families using linkage analysis followed by direct sequencing and identified pathogenic variants in the *WNT10B* gene (9, 24, 25). The Clinical features of affected members in these families exhibited SHFM phenotype, which is inherited in the form of autosomal recessive pattern. The SHFM features observed in our patients show similarities to those reported previously (8, 9, 24, 26). Detailed clinical comparisons of our patients with that reported earlier are presented in Table 2.

During organogenesis, Wnt signaling plays a significant role in proximal-distal outgrowth as well as dorso-ventral patterning of limb formations (27). Wnt signaling is essential in cartilage, bone, muscle and joint development (28). Other WNT genes such as *WNT3, WNT4, WNT6, WNT7A, WNT7B, WNT9B, WNT10A*, and *WNT16* as well as *WNT10B* show higher expression throughout the limb bud ectoderm in all phases of mouse limb formation with the exception of the apical ectodermal ridge where Wnt10b expression is only seen at embryonic day 11.5 (E11.5) (29, 30).

A few individuals were described carrying *WNT10B* variants exhibiting developmental tooth alterations, low bone mass or obesity but causality was not established (25, 26, 31, 32). Such phenotypes were not observed in our patients. Until now, 20 different types of (missense, nonsense, splice site, and frameshift) mutation have been identified in the *WNT10B* gene causing different human disorders including obesity, dental anomalies, and SHFM (Table 4) (HGMD: http://www.hgmd.org). Among the 20 HGMD reported mutations, only 12 mutations showed associations with SHFM anomalies. In line with previous reports, the *WNT10B* mutation (p.Cys366^*^) identified in this study is predicted to affect the development of the hands and feet resulting in SHFM type 6. Our findings support the vital function of *WNT10B* in the human skeleton development.

**Table 4:** Mutations identified in *WNT10B* gene and associated HGMD disorders identified in different ethnic groups.

**Transcript ID**	**Gene name**	**DNA variation**	**Protein variation**	**Mutation location**	**Mutation type**	HGMD^*^ reported phenotype	**Ethnicity of reported families**
NM_003394.4	*WNT10B*	c.1098C>A	p.Cys366^*^	Exon 5	Nonsense	Split Hand/Foot Malformation (Present study)	Pakistan
NM_003394.4	*WNT10B*	c.265G>A	p.Asp89Asn	Exon 3	Missense	Dental Anomalies, isolated	Thailand
NM_003394.4	*WNT10B*	c.475G>C	p.Ala159Pro	Exon 4	Missense	Dental anomalies, isolated	Thailand
NM_003394.4	*WNT10B*	c.994C>T	p. Arg332Trp	Exon 5	Missense	Split hand/foot malformation	Turkey
NM_003394.4	*WNT10B*	c.569C>G	p.Pro190Arg	Exon 4	Missense	Oligodontia	Chinese
NM_003394.4	*WNT10B*	c.632G>A	p.Pro211Gln	Exon 4	Missense	Oligodontia	Chinese
NM_003394.4	*WNT10B*	c.661C>T	p.Arg221Trp	Exon 4	Missense	Split hand/foot malformation	United State
NM_003394.4	*WNT10B*	c.767G>A	p.Cys256Tyr	Exon 5	Missense	Obesity	European
NM_003394.4	*WNT10B*	c.786G>A	p.Trp262^*^	Exon 5	Nonsense	Oligodontia	Chinese
NM_003394.4	*WNT10B*	c.849C>A	p.Ile283Ile	Exon 5	Missense	Oligodontia	Chinese
NM_003394.4	*WNT10B*	c.851T>G	p.Phe284Cys	Exon 5	Missense	Oligodontia	Chinese
NM_003394.4	*WNT10B*	c.986C>G	p.Thr329Arg	Exon 5	Missense	Split hand/foot malformation	Pakistan
NM_003394.4	*WNT10B*	c.986C>A	p.Thr329Lys	Exon 5	Missense	Split hand/foot malformation	United State
NM_003394.4	*WNT10B*	c.1052G>A	p.Arg351His	Exon 5	Missense	Split hand/foot malformation	Thailand
NM_003394.4	*WNT10B*	c.1087C>T	p.Arg363Cys	Exon 5	Missense	Split hand/foot malformation	Thailand
NM_003394.4	*WNT10B*	c.695_697delACA	p.Asn232del	Exon 4	Frameshift	Split hand/foot malformation	Indian
NM_003394.4	*WNT10B*	c.458_461dupAGCA	p.D115Afs^*^47	Exon 4	Frameshift	Split hand/foot malformation	Switzerland
NM_003394.4	*WNT10B*	c.293_299dupAGGGCGG	–	Exon 3	Frameshift	Split hand/foot malformation	Pakistan
NM_003394.4	*WNT10B*	c.300_306dupAGGGCGG	p.Leu103Argfs^*^53	Exon 3	Frameshift	Split hand/foot malformation	Pakistan
NM_003394.4	*WNT10B*	c.338-1G>C	–	Intron 3	Splice site	Split hand/foot malformation	Saudi Arabia
NM_003394.4	*WNT10B*	c.1165_1168delAAGT	p.Lys338Glufs^*^36	Exon 5	Frameshift	Split hand/foot malformation	Pakistan

HGMD

* *, Human genome mutation database*.

## Conclusion

We have reported a novel sequence variant (c.1098C>A; p.Cys366*) in the *WNT10B* gene in a consanguineous Pakistani family presenting with SHFM type 6. This study further extended the spectrum of mutations in the *WNT10B* gene which might be helpful in proper molecular diagnosis and genetic counseling.

## Web Resources

1000 Genomes—http://www.1000genomes.org/;
Exome Variant Server—http://evs.gs.washington.edu/EVS/;
GnomAD—https://gnomad.broadinstitute.org;
dbSNP—http://www.ncbi.nlm.nih.gov/SNP/;
OMIM—http://www.omim.org/;
HGMD—http://www.biobase-international.com/products/hgmd

## Data Availability Statement

The raw data supporting the conclusions of this article will be made available by the authors, without undue reservation, to any qualified researcher.

## Ethics Statement

The study design and protocol were approved by the Institutional Review Board (IRB) at Quaid-i-Azam University Islamabad Pakistan, the Ethical Review Committee (ERC) of Peking Union Medical College (Beijing, China), and China Medical University (Shenyang, China). For minors, written informed consent was signed by their parents. Written informed consent was obtained from the individual(s), and minor(s)' legal guardian/next of kin, for the publication of any potentially identifiable images or data included in this article.

## Author Contributions

AK and RW participated in the design of the study, performed PCR, gene sequencing, and manuscript writing. AK, MU, MAA, and SH studied family, collected blood samples, and extracted DNA. MAn, WA, and MAl participated in manuscript preparation and XZ collected funds and supervised the study progress. All authors read and approved the final manuscript.

### Conflict of Interest

The authors declare that the research was conducted in the absence of any commercial or financial relationships that could be construed as a potential conflict of interest.
